# Biomechanically based Fu’s subcutaneous needling treatment for senile knee osteoarthritis: protocol for a randomized controlled trial

**DOI:** 10.1186/s13018-024-04878-7

**Published:** 2024-07-08

**Authors:** Hai Huang, Ruixuan Liu, Jieying Shao, Shiyang Chen, Jian Sun, Junxia Zhu

**Affiliations:** 1 The Second Clinical College of Guangzhou University of Chinese Medicine, Guangzhou, Guangdong China; 2 The Eighth Clinical Medical School, Guangzhou University of Chinese Medicine, Guangzhou, Guangdong China; 3 Clinical Medical College of Acupuncture & Moxibustion and Rehabilitation, Guangzhou University of Chinese Medicine, Guangzhou, Guangdong China; 4 Baiyun Hospital of The First Affiliated Hospital of Guangzhou University of Chinese Medicine, Guangzhou, Guangdong China

**Keywords:** Fu’s subcutaneous needling, Knee osteoarthritis, Gait analysis, Muscle elasticity

## Abstract

**Introduction:**

Fu’s subcutaneous needling (FSN) is a new type of acupuncture that uses subcutaneous tissue to oscillate from side to side to improve muscle pathology status and can be effective in treating Knee osteoarthritis. Nonetheless, whether the clinical effect is similar to that of most commonly used drugs is unclear. Thus, this study aims to determine the pain-relieving effect and improvement in the joint function of the FSN therapy by comparing it with that of a positive control drug (celecoxib). Furthermore, this clinical trial also aims to evaluate the effect of FSN on gait and lower limb muscle flexibility, which can further explore the scientific mechanisms of the FSN therapy.

**Methods and analysis:**

This study is a randomized, parallel-controlled, single-center prospective clinical study that includes 60 participants, with an FSN group (n = 30) and a drug group (n = 30). The Fu’s subcutaneous needling (FSN) group undergo the FSN therapy 3 times a week for 2 weeks, while the drug group receives 0.2 g/day oral celecoxib for 2 weeks, with a follow-up period of 4 weeks after the completion of treatment. The primary outcome is the difference in the visual analog scale score after 2 weeks of treatment compared with baseline. The Western Ontario and McMaster Universities (WOMAC) Osteoarthritis Index, joint active range of motion test, three-dimensional gait analysis, and shear wave elastic imaging technology analysis in lower limb muscles are also performed to demonstrate clinical efficacy.

**Ethics and dissemination:**

The trial is performed following the Declaration of Helsinki. The study protocol and consent form have been approved by the Ethics Committee of Guangdong Provincial Hospital of Chinese Medicine. All patients will give informed consent before participation and the trial is initiated after approval. The results of this trial will be disseminated through publication in peer-reviewed journals.

*Trial registration number*: NCT06328153.

**Supplementary Information:**

The online version contains supplementary material available at 10.1186/s13018-024-04878-7.

## Introduction

Knee osteoarthritis (KOA) is one of the most common types of osteoarthritis, with clinical manifestations of knee pain, stiffness, and limited function, which in severe cases leads to bone deformity of the knee [1, 2]. Globally, nearly 30% of people over 45 years of age have imaging manifestations of KOA, and approximately half of them are symptomatic [3]. In China, the prevalence of symptomatic KOA requiring medical consultation is as high as 8.1% [4]. Apparently, KOA poses a considerable burden to both patients and the national healthcare system. Age is an essential risk factor for the development of KOA, and the incidence increases with age and peaks in the elderly population after the age of 65 years [5, 6]. The pathogenesis of the disease is currently thought to be mainly related to abnormal mechanical factors, including abnormal joint mechanics, muscle dysfunction, gait abnormalities, and disturbances in bone alignment, which lead to biochemically mediated biological effects [7, 8].

Studies have shown that abnormal skeletal muscle function in the lower limbs, especially weakening of the quadriceps, increases the load on the knee joint, which is closely related to this disease [9]. Skeletal muscles can absorb loads. In normal physiology, the biomechanical effect of the muscles around the knee joint on the joint is in balance with the external loads acting on the knee joint as a whole, whereas in the pathological state, due to the dysfunction of the muscles, the mechanical loads acting on the knee joint are in a state of imbalance, which leads to an abnormal patellar movement trajectory. With a decrease in the stability of the joint, it provokes or exacerbates inflammatory pathology of the articular cartilage and dysfunction of the knee joint [9–12]. Lower limb muscle weakness has been reported to precede the onset of joint pain and imaging changes in patients with senile knee osteoarthritis, and diminished muscle strength is positively correlated with disease severity [13, 14]. It was also found that by improving the mechanical properties of the lower limb muscle, the knee adduction moment (KAM) and medial compartmental pressure of the knee could be reduced, thus counteracting the abnormal external loads acting on the knee joint and ultimately achieving a therapeutic effect on KOA [10]. It is clear that improving the condition of the relevant lower limb muscles, especially the quadriceps, may play an important role in the prevention and treatment of senile knee osteoarthritis.

Currently, acupuncture has been increasingly used in the practice of KOA treatment, effectively relieving the symptoms of pain and joint stiffness [15, 16]. A large-sample, multicentre study found that electroacupuncture treatment for KOA achieved a response rate of 60.3% at Week 1, and demonstrated satisfactory long-term effects at Week 26 [17]. Fu’s subcutaneous needling (FSN) is an emerging acupuncture modality, characterized as safe, eco-friendly and efficient. It has been used in clinical practice for more than 20 years. The practitioner uses a specially designed needle to perform horizontal reciprocal swaying within loose connective tissues under the skin, in conjunction with the Reperfusion Approach. FSN therapy is distinguished by its rapid onset of effect [18]. A meta-analysis showed that FSN significantly treated KOA and had a faster onset of effect than did manual acupuncture, drugs, and herbal medicine [19]. The only target objects for FSN are muscles under pathological tension. It has been demonstrated that the reciprocal Swaying Movement of the FSN in the loose connective tissue and the Reperfusion Approach of muscle contraction-diastole elevate the perfusion of vascular blood flow through the interosseous space [20]. This restores healthy muscle elasticity [21] and further restores joint stability of the knee, thereby having a therapeutic effect on KOA. We hypothesized that this is the reason why FSN is effective.

Our preliminary study revealed that the modulus of elasticity of the lateral femoral muscle was significantly higher on the affected side than on the healthy side in patients with KOA (*p* < 0.01). It was also found that FSN significantly improved symptoms and signs while reducing the modulus of elasticity of the lower limb skeletal muscles (vastus lateralis, tibialis anterior, and gastrocnemius) of KOA patients (*p* < 0.05), and a related paper is being prepared for publication. Previously, two studies have suggested that FSN therapy applied for the treatment of KOA effectively relieved pain [22, 23]. However, both studies only applied pain-related measures or function-related measures as indicators of efficacy. Few studies have focused on a biomechanical point of view. There are also few published clinical studies that have used objective measures other than scales, such as ultrasonography or imaging. Therefore, the objective of this randomized clinical trial is to investigate the clinical effects and biomechanical mechanisms of FSN for the treatment of elderly patients with knee osteoarthritis. We hypothesized that an intervention programme using FSN for 2 weeks, 3 times per week, would produce better improvements in pain as well as physical function of the knee compared to a positive control group taking celecoxib. Furthermore, this clinical trial also aims to evaluate the ability of FSN to improve gait and lower limb muscle flexibility, which may further help explore the mechanism of FSN therapy.

## Method and analyses

### Study design

The study will be conducted from May 2024 to March 2026 at Guangdong Provincial Hospital of Chinese Medicine and will be performed according to the Declaration of Helsinki.

This is a randomized controlled, parallel, single-center prospective clinical trial. The study has been approved by the Ethics Committee of Guangdong Provincial Hospital of Chinese Medicine (Ethics registration ID: BF2022-101-01) and was registered on the clinical trial platform (ClinicalTrials.gov ID: NCT06328153; Protocol ID: BF2022-101).

### Study setting

**Fig. 1:**
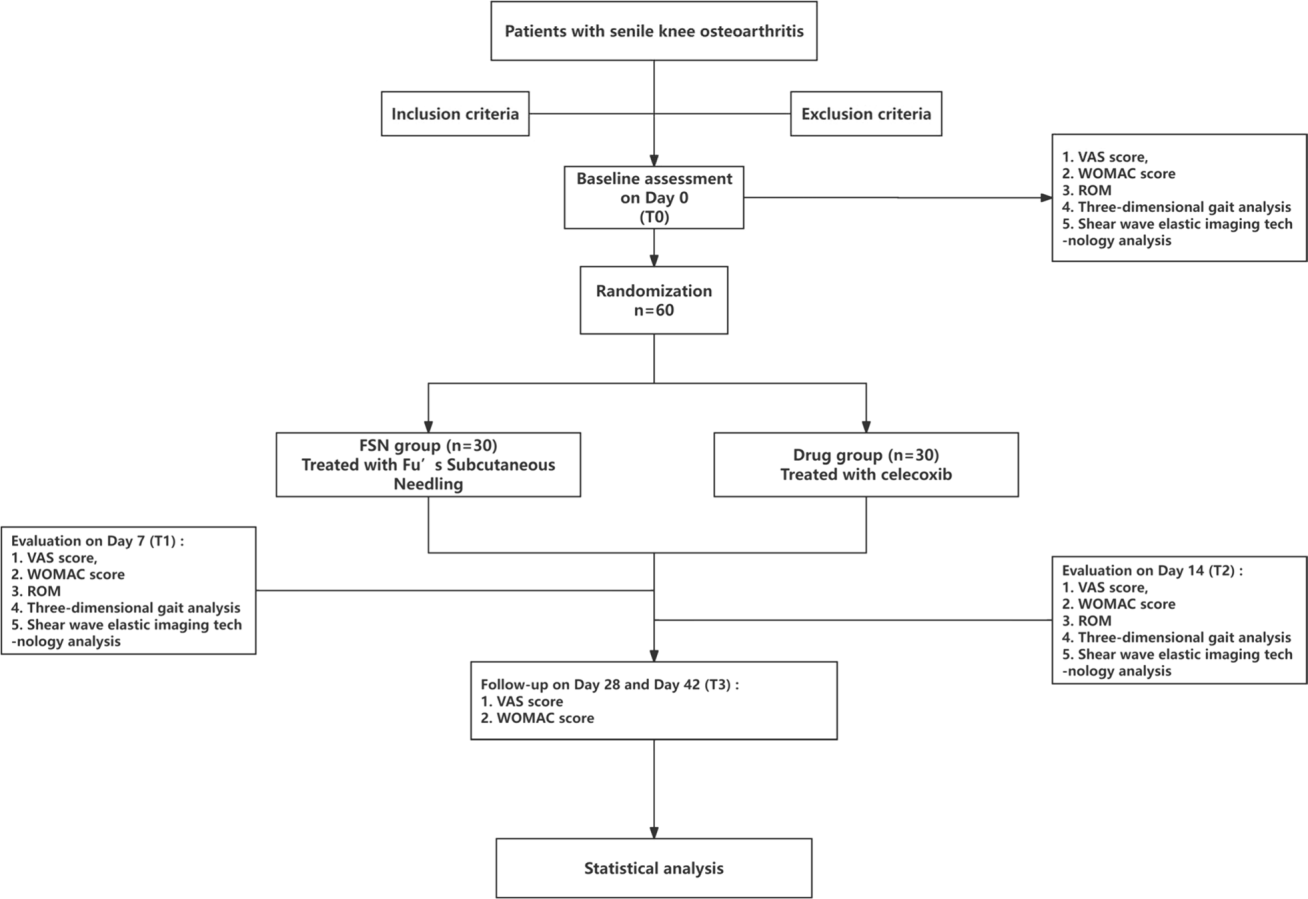
Planned study protocol flowchart

Participants are recruited from the outpatient department of Guangdong Provincial Hospital of Chinese Medicine. Eligible and consented participants are randomly assigned at a ratio of 1:1 to receive treatment in the FSN group (n = 30) or drug group (n = 30) for 2 weeks. Participants are assessed at baseline and on the 7th, 14th, 28th, and 42nd days after the start of the intervention. The design of the trial is summarized in Fig. 1.

### Participants

#### Inclusion criteria

Participants who meet the diagnostic criteria for KOA according to the Guidelines for the Diagnosis and Treatment of Osteoarthritis (2018 Edition) by Osteoporosis Group of the Chinese Orthopaedic Associationg [24] and the diagnostic criteria for KOA formulated by the American College of Rheumatology [25] are eligible to participate when they also meet the following criteria:

1. The patients are aged between 60 and 80 years.
2. BMI < 28.
3. Kellgren–Lawrence grade 1–3.
4. Knee pain score > 3 on a 10-point numerical rating scale (VAS).
5. Voluntary and capable of signing the informed consent form.

#### Exclusion criteria

1. Non-primary knee osteoarthritis, such as gouty arthritis and traumatic arthritis.
2. According to the above guidelines, the affected knee severely deformed, either clinically or radiologically.
3. Patients who have taken analgesics or Non-steroidal Anti-Inflammatory Agents (NSAIDs) orally or topically on the affected knee within the last month; who have received adjuvant treatment for the affected knee joint (e.g., physical therapy, acupuncture, massage, etc.) within the last 3 months; or who have a history of knee surgery in the past 6 months.
4. With other diseases that may cause lower limb pain, such as lumbar disc herniation and lumbar spinal stenosis.
5. With lower extremity vascular diseases.
6. With Severe systemic or lower limb local skin diseases.
7. With any unstable medical disease (e.g., acute cardiac or cerebrovascular disease, poorly controlled diabetes, etc.) or psychiatric illness.
8. With contraindications to NSAIDs, which include postcoronary artery bypass graft surgery, active peptic ulcer, severe heart failure, etc.

### Sample size calculation

The main outcome measure of this study is the decrease in VAS score from baseline during the 2-week treatment period. FSN, an emerging acupuncture method, has been studied in randomized controlled trials [22, 23]. However, both of these previous studies used superiority testing, which cannot provide an appropriate reference for the sample size calculation of our study. Therefore, we refer to a randomized controlled trial that compared electroacupuncture with an NSAID for treating KOA [26]. We assume that the effect sizes of interventions (FSN and celecoxib) in this trial were similar to those (acupuncture and diclofenac applied for interventions) in the aforementioned study. The results of the above study showed that the VAS score decreased by 48.24 ± 3.59 mm from baseline after 4 weeks of electroacupuncture treatment, and by 32.99 ± 3.94 mm from baseline after 4 weeks of oral administration of diclofenac. The actual difference in the VAS score between the two interventions after 4 weeks of treatment was 15.25 mm, with a combined standard deviation of 3.85 mm. However, since the course of treatment in our trial is half of that in the aforementioned study, appropriate adjustments for the expected difference in the VAS score are needed. Based on our clinical experience, the efficacy and the treatment period cannot be considered to increase in full equivalence, and since the 2-weeks intervention course is relatively short, we would not expect the cumulative efficacy to be half that of the 4-weeks intervention in the aforementioned study.

Therefore, to compare the efficacy of FSN and celecoxib, we assume that the difference in two groups’ means of the changes in the VAS score is 6.5 mm after 2 weeks of treatment, which is slightly less than half the difference observed in the reference trial [26] after 4 weeks of intervention. Assuming a 1:1 ratio of participants in the 2 groups, the NIM (Non-Inferiority Margin) is defined as 10 mm. A two-sided test with a significance level (α) of 0.05 and a power(1-β) of 0.80 is applied. With a possible dropout rate of 10%, using the PASS 15.0 software, 60 participants shall be included in this study.

### Randomization and blinding

Eligible and consenting participants are randomly assigned to the FSN group (n = 30) or the drug group (n = 30) for the clinical study at a 1:1 ratio through a central randomization system. The randomization sequences are generated by staff using the SAS program. Patient serial numbers, random numbers, and grouping results are assembled into random assignment cards that are then sealed in disposable opaque envelopes. The cards in the envelopes have serial numbers that match the numbers on the envelopes. The random assignment cards are kept in a special place. When eligible participants enter the study, envelopes with the same serial numbers are opened in the order of the participants. Participants are grouped and treated according to the cards they obtain.

Due to the specific nature of the needling operation, the participants and practitioners involved in this study are not blinded. However, outcome assessors are blinded to the group assignments and instructed not to communicate with participants regarding the intervention to ensure objectivity in outcome evaluations. The researchers who are responsible for data collection and statistical analyses are also blinded to the assigned tasks to minimize bias in the reporting of subjective results.

### Interventions

Participants’ gender, age, marital and childbearing history, family history, allergy history, smoking and drinking history, past medical history, drug history, and other general information are collected before the formal intervention. The participants’ baseline data, such as weight and BMI, are observed. Visual analogue scale (VAS) measurements, Western Ontario and McMaster Universities (WOMAC) index measurements, joint active range of motion tests, three-dimensional gait analyses, and shear wave elastic imaging technology analyses are performed to comprehensively evaluate the pain level, knee joint movement status and system function, as well as the lower limb skeletal muscle elastic modulus. To ensure participants adhere to protocol requirements for timely FSN attendance or scheduled dosing of celecoxib, education is provided and schedules are distributed. Participants are also instructed to avoid other KOA treatment that may affect efficacy evaluation, such as oral or topical analgesics, manual acupuncture and massage.

### FSN therapy

**Fig. 2:**
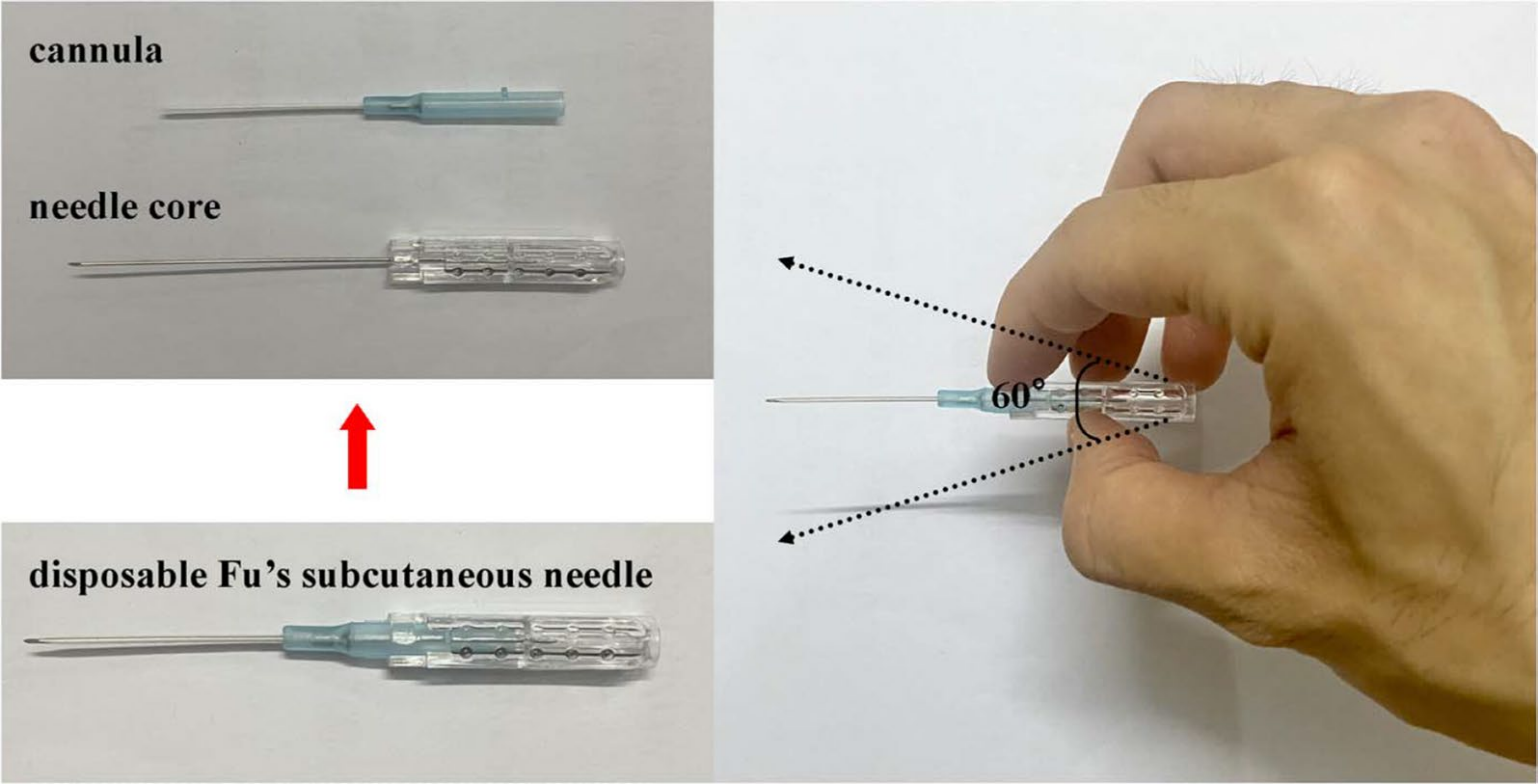
A disposable FSN needle consists of a cannula and a needle core. The practitioner holds the needle handle and manipulates it in a fan-shaped pattern at an angle of approximately 60°

**Fig. 3:**
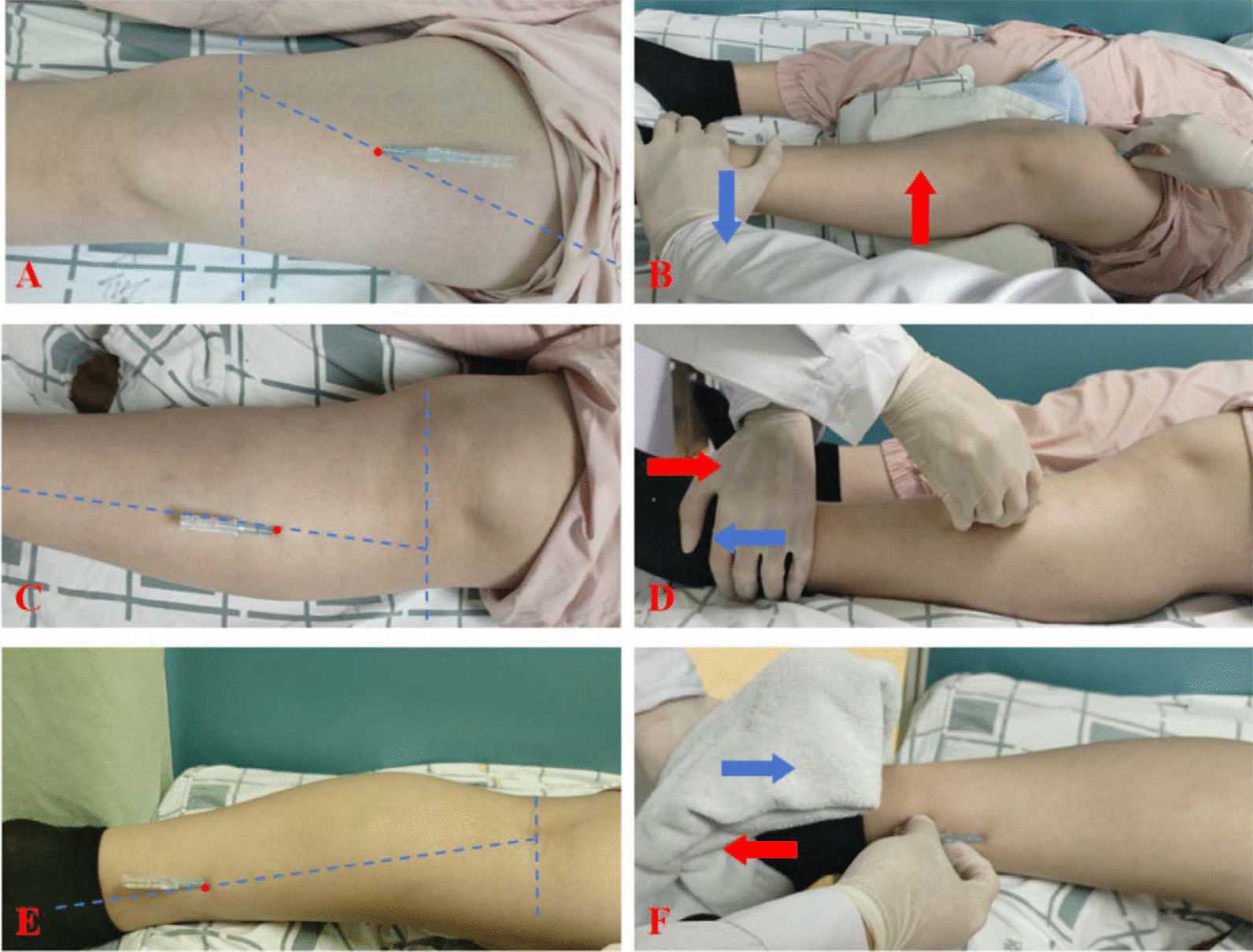
FSN entry point and Reperfusion manoeuvre. **A** Quadriceps muscle entry point: 1/3 below the line between the anterior superior iliac spine and the medial superior border of the patella; **B** Quadriceps muscle Reperfusion manoeuvre; **C** Tibialis anterior muscle entry point: 1/3 above the line between the caput fibulae point and the first metatarsal bone; **D** Tibialis anterior muscle Reperfusion manoeuvre; **E** Gastrocnemius muscle entry point: 1/3 below the line between the medial ankle and midpoint of the popliteal fossa; **F** Gastrocnemius muscle Reperfusion manoeuvre. *Notes*: The red arrow shows the participant’s direction of force and the blue arrow shows the instructor’s direction of force

Operation of the FSN follows the KOA’s Fu’s Subcutaneous Needling Treatment Code of Practice [27]. Participants first assume the supine position. After routine disinfection, a disposable Fu’s subcutaneous needle (Nanjing Paifu Medical Technology Co., Ltd., Jiangsu, China, Fig. 2) is inserted parallel into the subcutaneous loose connective tissue around the pathological tight muscles (gastrocnemius muscle, tibialis anterior muscle, and quadriceps femoris muscle). The protruding part of the hose holder is fixed to the card slot after the needle is completely inserted. Confirming that the participants have no pain, the index and ring fingers alternate back and forth in a smooth, soft, fan-like swaying movement. The fan angle is approximately 60°, and a total of 45 round trips are performed in 30 s. Swaying Movement is accompanied by Reperfusion Approach: 20 sways along with 10 s of Reperfusion Approach are performed as a set of procedures. For each target pathological tight muscle, 2–3 sets of the above procedures are required. The Reperfusion Approach for different pathological tight muscles are as follows: (1) for the gastrocnemius muscle, the participant is placed in the prone position, with the foot extended out of the bed. A towel is placed on the soles of the feet, and the participant is instructed to resist resistance by plantar flexion of the ankle joint; (2) for the tibialis anterior muscle,the participant is placed in the supine position, and the ankle is dorsiflexed to resist counterdirectional forces from the instructor, (3) for the quadriceps muscle,the participant is placed in the supine position. With a foam shaft or other support made of soft material placed in the popliteal fossa, the knee joint is then forcefully extended against resistance from the instructor. The FSN group is treated 3 times a week for 2 weeks. Prior each FSN treatment day, participants are contacted by telephone in advance to schedule specific sessions and are reminded to attend. (Fig. 3).

### Oral celecoxib

Participants in the drug group receive 200 mg of oral celecoxib (a capsule) daily for 2 weeks. They are required to mark prepared schedules after each dose. After the seventh and fourteenth doses, participants return to the research center with their schedules for review. They are closely followed up during the administration of the drug, and the treatment will be discontinued immediately in case of adverse events. If necessary, appropriate symptomatic treatments, such as acid suppressing, protection of the gastric mucosa, anti-vertigo medication, etc., will be administered until the adverse events subside.

### Outcomes

The evaluators detect the outcomes at baseline, on day 7, day 14, and during the follow-up period (day 28 and day 42 after the start of the treatment) (Table 1).

**Table 1:** Study schedule

Period	Admission day	Baseline assessment	Evaluation on Day 7	Evaluation on Day 14	Follow-up period
Time point	− T1	T0	T1	T2	T3
Enrolment					
Eligibility	⭕				
Informed consent	⭕				
Allocation	⭕				
Interventions					
FSN			⭕	⭕	
Celecoxib			⭕	⭕	
Evaluation					
VAS score		⭕	⭕	⭕	⭕
WOMAC score		⭕	⭕	⭕	⭕
ROM		⭕	⭕	⭕	
3D gait analysis		⭕	⭕	⭕	
Shear wave elastic imaging					
Technology analysis		⭕	⭕	⭕	
Adverse events			⭕	⭕	⭕

#### Primary outcome

KOA is characterized by knee pain as its predominant symptom. Visual Analog Scale (VAS) scores, ranging from 0 to 100 mm, are utilized to objectively quantify pain levels. The primary outcome of this study is the difference in VAS scores between day 14 and baseline measurements. Participants are instructed to indicate the severity of their knee pain by marking a point on a continuous horizontal line segment, with 0 mm representing minimal pain and 100 mm indicating the most severe pain [28].

#### Secondary outcomes

**Fig. 4:**
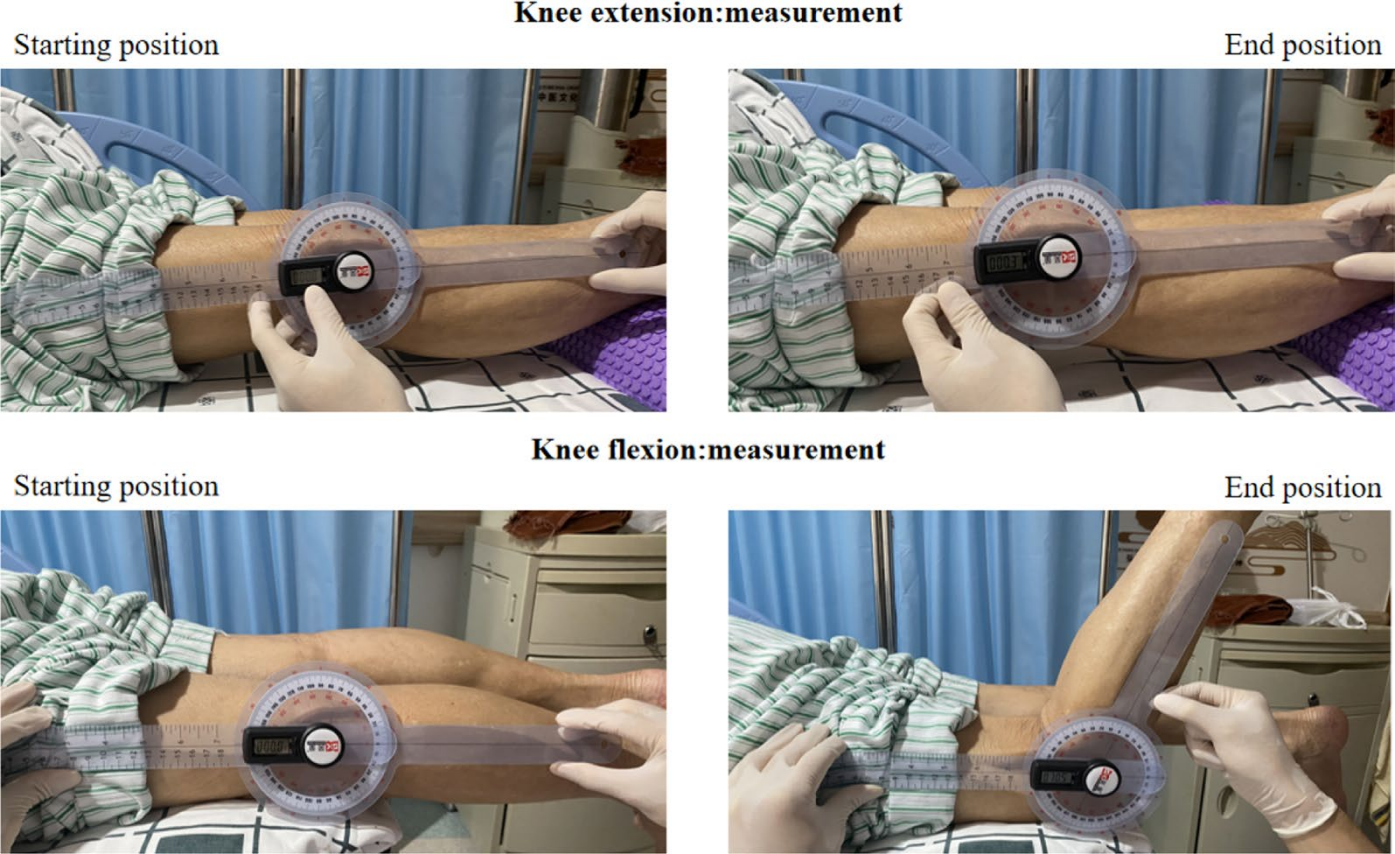
Measurement of ROM

**Fig. 5:**
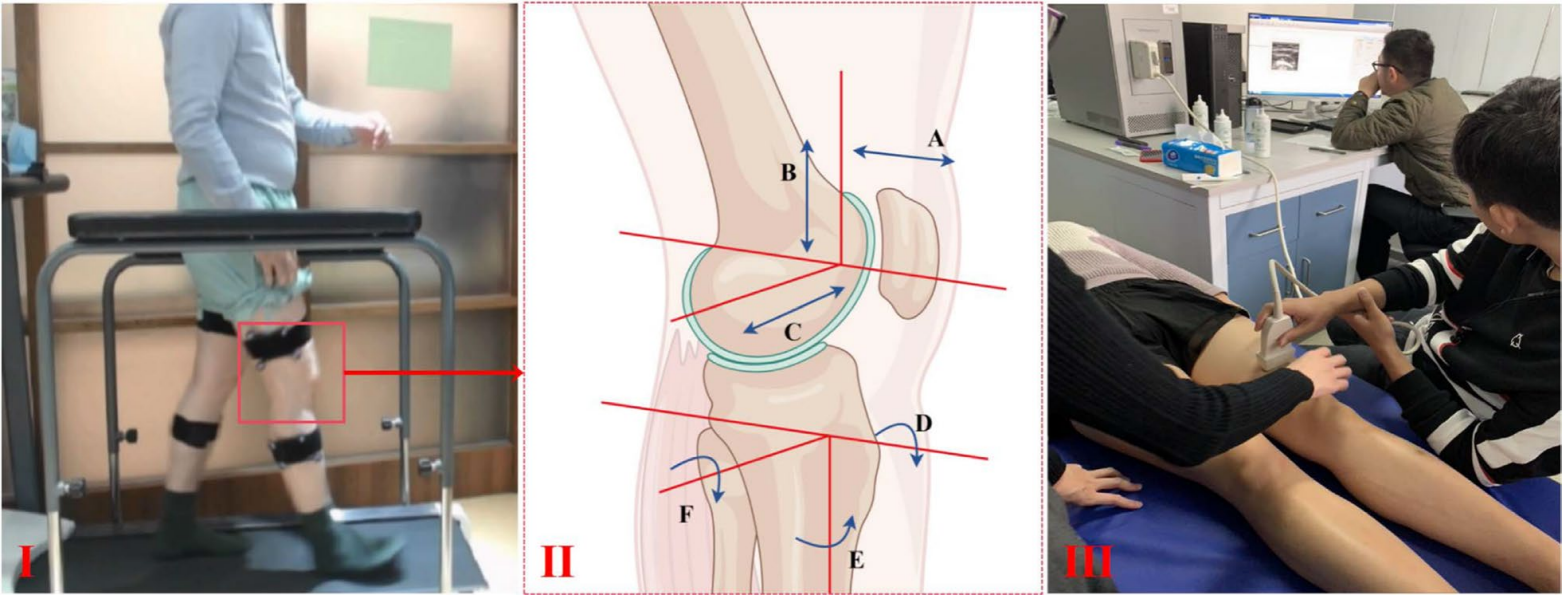
Schematic of three-dimensional gait analysis and shear wave elastic imaging technology analysis. (I) Three-dimensional gait analysis of the participants; (II) three-dimensional data of the knee; (III) shear wave elastic imaging technology analysis performed by doctors. For the labels in II. **A** anterior/posterior displacement; **B** superior/inferior displacement; **C** internal/external displacement; **D** internal/external flip angle; **E** external/internal rotation angle; **F** flexion/extension angle. (By Figdraw https://www.figdraw.com/.)

1. **VAS:** We assess patients’ knee pain using the VAS at baseline, on day 7 and during the follow-up period as secondary outcome indicators.
2. **WOMAC:** The Western Ontario and McMaster Universities Arthritis Index (WOMAC) is widely used to assess the clinical manifestations of knee disorders. We assess the WOMAC scores at baseline, on day 7, day 14, and during the follow-up period. The WOMAC index consists of three sections including evaluation of pain (5 questions), stiffness (2 questions), and physical function (17 questions). A five-level scoring system is applied for each question: asymptomatic (0 point), mild (1 point), moderate (2 points), severe (3 points), and very severe (4 points). Participants complete the index according to their actual conditions. Higher scores indicate more severe symptoms [29]. The temporal evolution of WOMAC scores is analyzed within each group. The scores on each assessment day are also compared between the two groups.
3. **ROM:** Range of Motion (ROM) is evaluated to visually record the motion angle of knee joint. Active ROM, including flexion and extension, is measured at baseline, on day 7 and day 14. For active flexion ROM, the participant are positioned prone with knees at the bed’s edge and lower legs extended naturally. The participant then flexes the knees maximally. Thus, the flexion angle is recorded. For active extension ROM, the participant lies supine with lower limbs extended and ankles slightly elevated by a cushion. The participant then extends the knees maximally. Thus, the extension angle is recorded [30, 31]. Measurements are repeated three times and averaged. Generally, abnormal restriction of active ROM is associated with adhesion of joint cavity, contracture of tendons and muscle weakness. The improvement in active ROM indicates knee healing. Thus, active ROM is evaluated to compare the efficacy of the interventions. (Fig. 4)
4. **Three-dimensional Gait Analysis:** An Opti-Knee 3D motion analysis system equipped with Guangdong Provincial Hospital of Chinese Medicine is used to detect the motion data of the knee joint. The parameters of six degrees of freedom (6 DOFs) are collected and analyzed. The indicator is detected on day 0, day 7 and day 14. The knee joint exhibits complex motion, primarily resulting from the relative movement of the tibia and femur. Based on the principles of the anatomical coordinate system, a three-dimensional femur-tibia coordinate system is established. Thus, it allows the decomposition of the relative motion between the tibia and the femur into 6 DOFs. The DOFs rotating around the coordinate axes includes: internal/external rotation angle, internal/external flip angle, flexion/extension angle. The DOFs translated along the coordinate axes includes: anterior/posterior displacement, superior/inferior displacement, and internal/external displacement [32, 33]. During practice, the participant is asked to undergo approximately 2–3 min of acclimatization. After the bony marks on the body surface are ready for positioning, the participant walks continuously on the exercise board in a natural habitual way at a constant speed of 3.0 km/h. Subsequently, the system collects gait data of 6 DOFs at a frequency of 60 frames/s and an acquisition time of 15 s. The temporal evolution of gait parameters is depicted through curves, and the data are then exported in the form of single-cycle gait data (Fig. 5). In our results, we concentrate on three primary aspects: a comparison of 6 DOFs of the affected knee and those of the healthy side; the evolution of 6 DOFs over the treatment course within the same group; and a comparison of 6 DOFs discrepancies between the two groups. By modeling the participants’ habitual flat walking patterns, we investigate whether the kinematic deficiencies of the affected knee demonstrate an improvement post-treatment, or if the kinematic characteristics of both knees exhibit a tendency to converge.
5. **Shear Wave Elastic Imaging Technology Analysis:** The mean elastic modulus serves as an indicator of muscle tone, which is associated with knee stiffness. We evaluate the Young’s modulus of major muscle groups of knee joint at baseline, on day 7 and day 14. A Supersonic Aixplorer Color Doppler Ultrasound Diagnostic Unit equipped with Guangdong Provincial Hospital of Chinese Medicine is used. Lying flat on the bed, the participant is instructed to relax all body muscles and to straighten both legs naturally. First, 2D ultrasound is used to identify the major muscles. The device is then switched to the SWE mode, with an L15-4 line array probe and a frequency of 4–12 MHz. With the ultrasound set to muscle examination mode, we sequentially measure the vastus intermedius, rectus femoris, vastus lateralis, and tibialis anterior muscles. The probe is lightly placed at the midpoint of the examined muscle belly, and it is parallel to the direction of the muscle fibers. With no pressure applied, skeletal muscles can be regarded as having three layers of depth: the upper layer, the middle layer, and the lower layer. In the ultrasound display, the middle layer is selected to place the sampling box for quantitative analyses. After 5 s, the image is frozen as the shear wave pattern in the box is stable and uniform. The Q-BOX function is launched, with the measurement area set as a 5-mm circle. The system then automatically calculate the mean value of Young’s modulus for the muscle tissues in the Q-BOX (Mean). Each frozen image with the calculation is repeated 3 times, and the mean values of 2 qualified sampling boxes are taken for statistical analyses (Fig. 5). Prior studies have suggested that the higher the value of the elastic modulus of the muscles around the knee, the higher the muscle tone and the tighter the epimysia, which results in restriction to the contraction of muscle fibers and function of the knee joint [34–36]. However, other studies have either failed to find significant results [37] or have reached contrary conclusions [38]. In response to these mixed findings, we investigate the elastic modulus of the major muscles along the force line of the lower extremity, aiming to provide credible evidence.

#### Assessment of safety

To evaluate the safety of treatment, the proportions of participants with adverse reactions in each group (T) are calculated. Adverse events are recorded and handled throughout the treatment and follow-up periods. According to previous literature and clinical experience, complications of FSN include subcutaneous hematoma, subcutaneous hemorrhage, and acupuncture syncope [39–41]. A clinical study reported that 5.36% of participants experienced mild subcutaneous hemorrhage or hematoma at the operating site of FSN treatment [39]. One RCT reported a 3.84% incidence of acupuncture syncope and a 3.84% incidence of subcutaneous hematoma in the FSN group [40]. In another RCT, 2.22% of participants in the FSN group experienced acupuncture syncope, while 4.44% had subcutaneous hemorrhage [41]. However, the clinical symptoms of these complications or adverse reactions were mild, and no serious cases were reported. For these minor complications, management strategies were generally simple, and patients recovered completely in a short time without sequelae. Regarding the potential risk of infection caused by FSN, no literature has clearly reported the occurrence, nor has any literature statistically analyzed the incidence. However, since the needle is inserted into the subcutaneous layer, there is a possibility of local skin infection even though the skin has been sterilized in advance. Should an infection occur, the needle is removed immediately. In consultation with the dermatology department, skin care, etiological testing, and appropriate antibiotics are administered if necessary. For the administration of celecoxib, possible complications and adverse effects include gastrointestinal reactions, renal insufficiency, rashes, etc. [42, 43]. The complications are recorded during medication and at follow-up visits, and reported to the the researchers immediately. Any complication is documented using a preset questionnaire, and appropriate measures are taken accordingly. If a more serious adverse reaction develops and the investigator considers the participant unfit to proceed with the study, the experimental interventions for this participant will be promptly terminated.

### Statistical analysis

All the statistical tests are conducted using a bilateral test, with *P* < 0.05 indicating a statistically significant difference. The Mean ± standard deviation is used for normally distributed measurement data. Non-normal data are presented as the median and interquartile range. Differences in data on outcome measures from baseline to each evaluation time point are analyzed using independent sample t tests or Mann–Whitney U tests, as appropriate, between groups; paired t tests or Wilcoxon tests are used to compare differences in the data for each outcome index at each evaluation time point within the group. In addition, the VAS and WOMAC evaluations are repeated 5 times in this study. Therefore, repeated measures analysis of variance are used for auxiliary analysis. Baseline data (including age, sex, BMI, etc.) are controlled for as confounding factors. Multiple imputation methods are adopted for the analyses of missing data. A generalized linear mixed model is further used for analyses of non-normal and partially missing data, as the interaction effects are also analyzed. The intention-to-treat analyses are performed for the outcomes. SPSS 25 software (IBM SPSS Statistics, New York, USA) is used for the data analyses. Data analyses are conducted by a data analyst who is unaware of allocation and treatment.

### Ethics

The research results of this project may be published in medical journals, but we will keep the patient’s information confidential by legal requirements. Unless required by relevant laws, the patient’s personal information will not be disclosed. When necessary, government management departments, hospital ethics committees, and their relevant personnel can access patient information according to regulations. Researchers or researchers who are authorized personnel are responsible for explaining the benefits and risks of participating in the trial to each patient, their legal representative, or a notary witness and should obtain written informed consent before the patient enters the trial. The original informed consent form signed and dated by all participants or their legal representatives, as well as the person presiding over the informed consent process, will be kept by the researchers.

### Dissemination and data sharing

The study will be reported according to the Consolidated Standards of Reporting Trials (CONSORT) statements. The results of their data will be disseminated to participants through interviews held with nurses. The final manuscript will be completed by the first two authors and the authorship of the final manuscript will depend on the actual contribution. The study results will be distributed using a broad dissemination strategy, including oral presentations at international meetings and publications in peer-reviewed international journals.

## Discussion

This study evaluates the effectiveness of FSN for the treatment of senile knee osteoarthritis compared to that of NSAIDs in terms of pain, self-reported function, gait, and muscle flexibility and assesses the differences in adverse events between the two interventions.

The main therapeutic objectives of KOA are to reduce pain, improve knee function, control arthritic symptoms, and enhance patients’ quality of life. In addition to weight loss and functional knee exercises, NSAIDs are consistently recommended by all guidelines as the first-line therapeutic agent to relieve pain [44, 45]. However, long-term application of NSAIDs carries the risks of gastrointestinal bleeding, cardiovascular adverse events, and nephrotoxicity. In addition, symptoms tend to recur once the drug is discontinued. For the elderly population, the risk of adverse reactions caused by NSAIDs is even greater. For example, the incidence of upper gastrointestinal bleeding is greater than that of other age groups, and the extent of the lesions is more severe [42, 43]. In the early or middle stage of KOA, to prevent the disease from progressing to an advanced stage that is difficult to reverse, clinical guidelines in various countries recommend treatments such as appropriate physiotherapy and muscle functional training, which aim to correct the pathological biomechanical imbalance of the affected knee joint [46, 47]. Therefore, in addition to medication, aggressive early intervention with complementary therapies for KOA may be of important.

As a newly emerging acupuncture method, FSN can also be used to treat KOA. Previous studies have suggested that FSN significantly relieves pain as well as joint stiffness and swelling and improves quality of life in patients with KOA [22, 23]. However, the biochemical mechanism by which FSN exerts its therapeutic effect remains unclear. The possible mechanism of action of FSN is that it improves blood circulation and muscle function. The significance of the Swaying Movement may be primarily related to mechanical, electrophysiological and biochemical effects in loose connective tissue [48, 49]. The Reperfusion Approach induces sudden compression and contraction of blood vessels in the involved muscles, followed by immediate vasodilation [50]. With blood circulation and tissue perfusion greatly accelerated, the vicious circle of Energy Crisis is interrupted [51]. These combined effects are believed to adjust the force lines of the lower limb. As the abnormal pressure on the cartilage decreases, it helps to maintain the stability of the knee joint, eventually allowing the forces on the joint to tend toward a normal balance.

The advantages of our study design are as follows: First, we rigorously designed and will conduct this trial to assess the effectiveness of FSN in treating KOA to provide evidence for decision-making and promotion of relevant complementary and alternative therapies; second, with the use of advanced instruments or equipment, we are the first to measure exploratory objective indicators to determine the mechanism of action of FSN in treating senile KOA from a biomechanical point of view, which can similarly provide clues for further research on FSN therapy and support its clinical application.

An evident limitation of this study is that, due to the inherent natures of acupuncture and medication, it is challenging to blind both the operators and the participants. This lack of blinding may increase the risk of bias. Consequently, the results of this study will be carefully evaluated and interpreted with a critical perspective. To minimize other potential sources of bias, the intervention operators, outcome assessors, data collectors, and data analysts act as independent investigators. Researchers, other than the intervention operators, shall not be aware of the group assignments of the participants. Additionally, instructions are provided throughout the trial to ensure that the participants undergo treatments and evaluations as planned, thereby avoiding potential implications of unplanned treatments on the outcome assessment. However, it is worth noting that the therapeutic effects of acupuncture are considered to involve both physical and psychological aspects [52]. Generally, the practice of acupuncture requires adjustment of patients’ mental state [53]. As a new method of acupuncture, the therapeutic effects of FSN are potentially diverse, encompassing direct physical effects and potential psychological effects. Thus, a placebo effect may actually be involved. The results of this study have the potential to inform further research. The combined curative effects of FSN could be analyzed from different perspectives, focusing separately on various mechanisms of action.

## Conclusion

The hypothesis of this study is that FSN therapy has a more significant and long-term effect on pain relief and improvement of joint function than conventional oral celecoxib in elderly patients with KOA. We aim to test this hypothesis through a rigorous clinical trial. We anticipate that the results of this study will provide guidance for the clinical application and promotion of FSN therapy in KOA. Furthermore, the preliminary analyses may help to further explore the rationale and potential indications of FSN.

### Supplementary Information

Below is the link to the electronic supplementary material.

Supplementary material 1.

Supplementary material 2.

Supplementary material 3.

Supplementary material 4.

Supplementary material 5.
