# Supplementary figures and images for: Biomechanically based Fu’s subcutaneous needling treatment for senile knee osteoarthritis: protocol for a randomized controlled trial
Source: J Orthop Surg Res. 2024 Jul 8;19:394. doi: 10.1186/s13018-024-04878-7 (PMC11232289; doi:10.1186/s13018-024-04878-7)

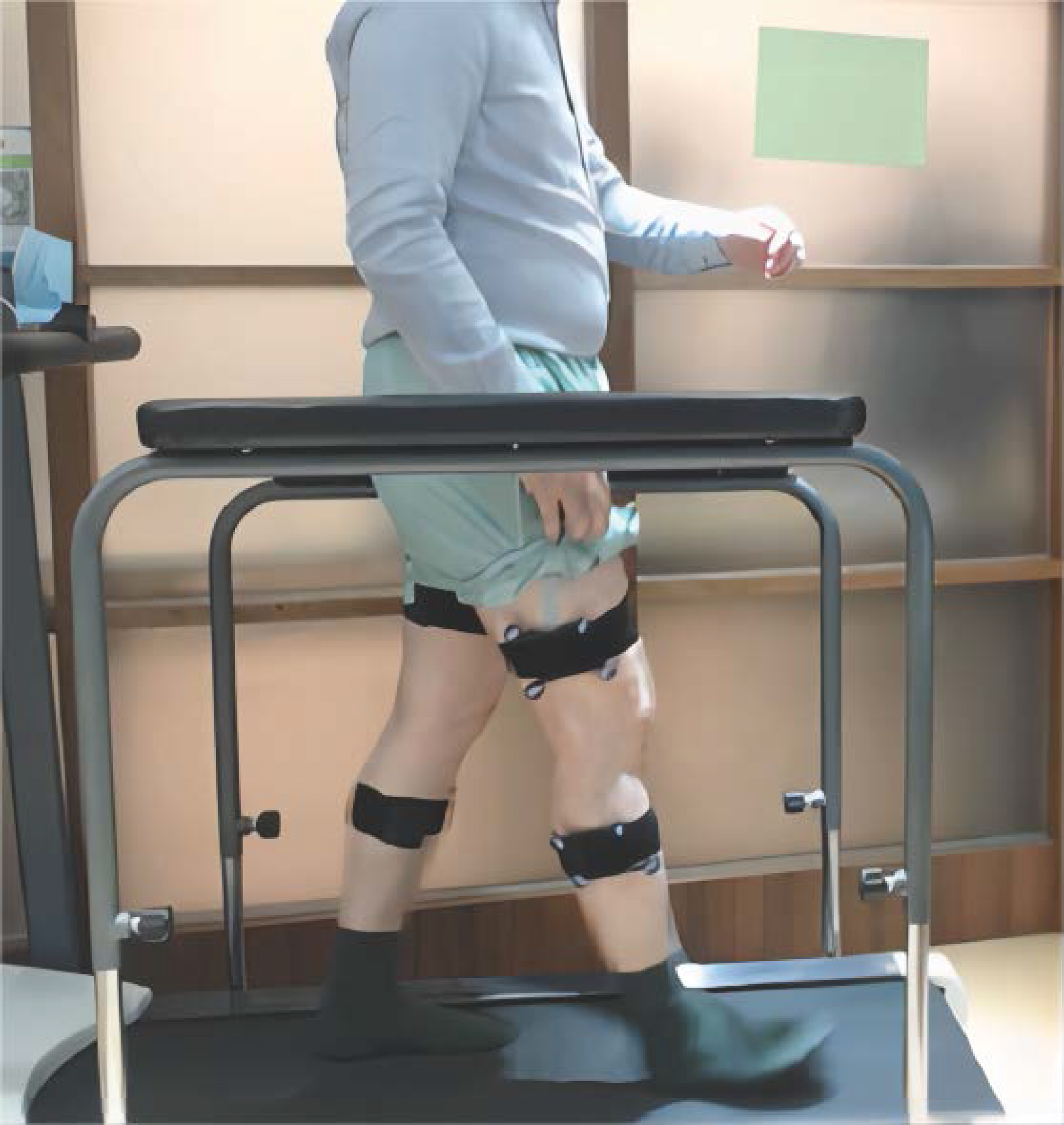

Supplement: Supplementary file 1 — Supplementary material 1. [file 13018_2024_4878_MOESM1_ESM.tiff]

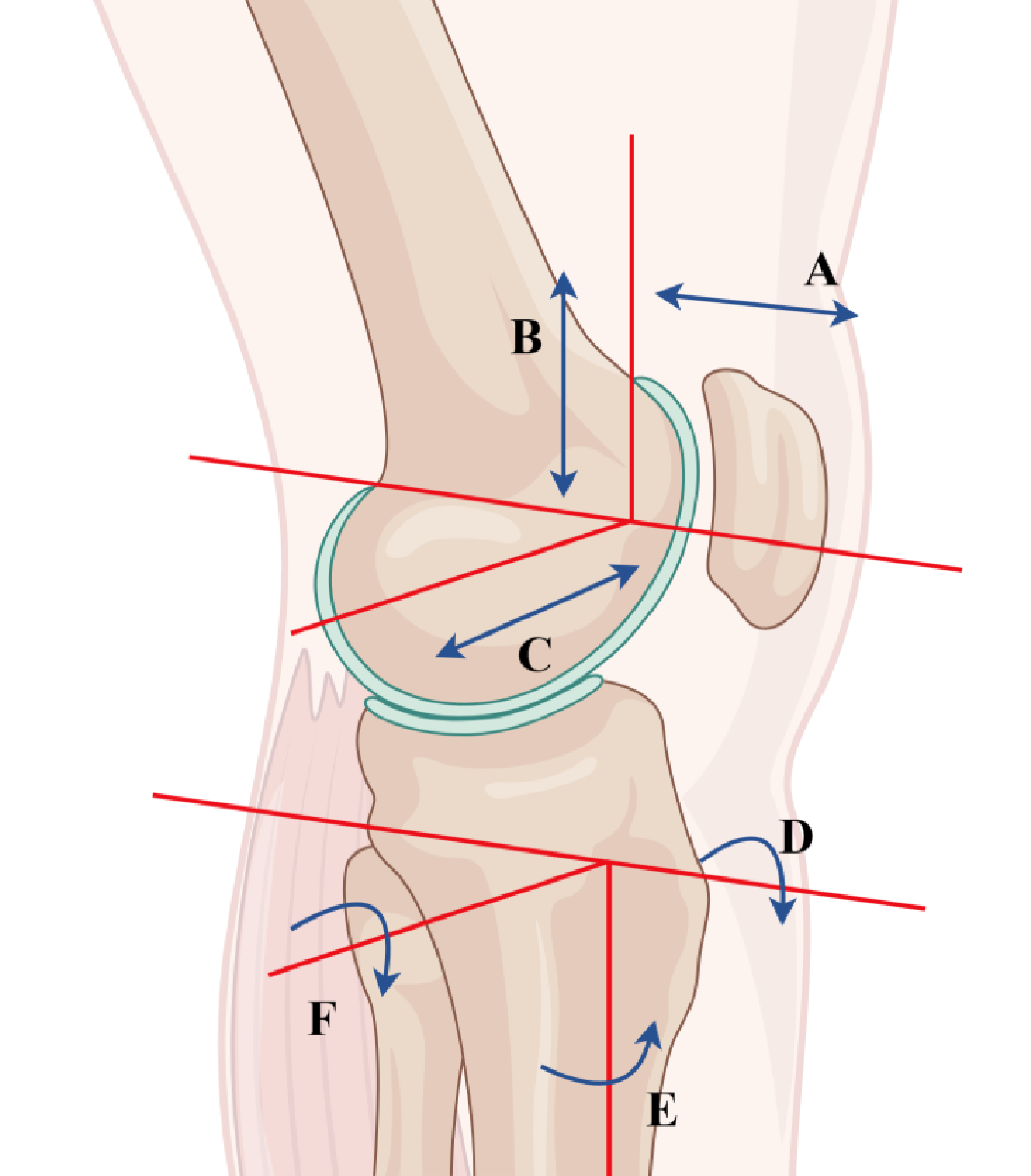

Supplement: Supplementary file 2 — Supplementary material 2. [file 13018_2024_4878_MOESM2_ESM.tif]

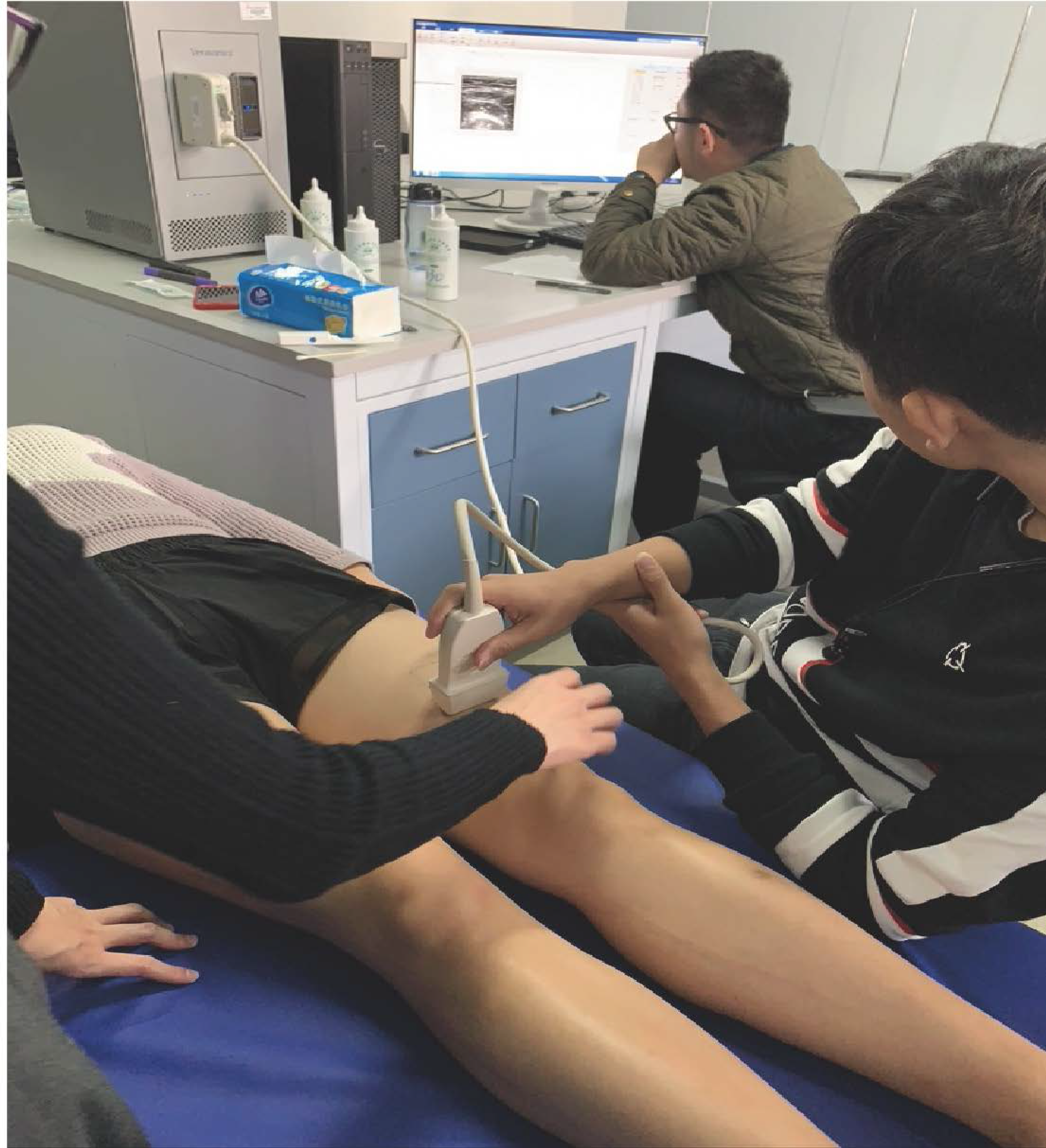

Supplement: Supplementary file 3 — Supplementary material 3. [file 13018_2024_4878_MOESM3_ESM.tif]

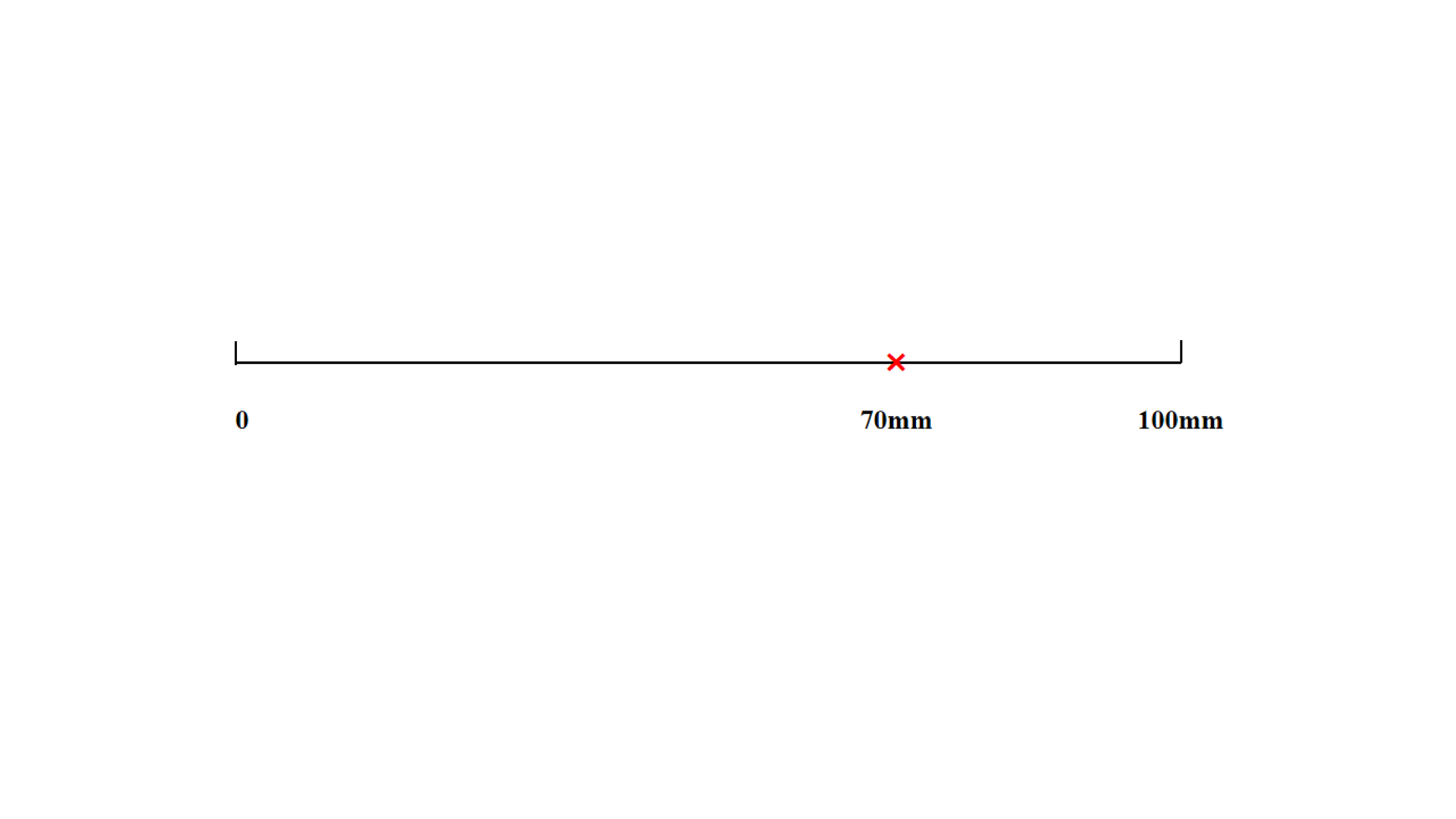

Supplement: Supplementary file 4 — Supplementary material 4. [file 13018_2024_4878_MOESM4_ESM.tif]

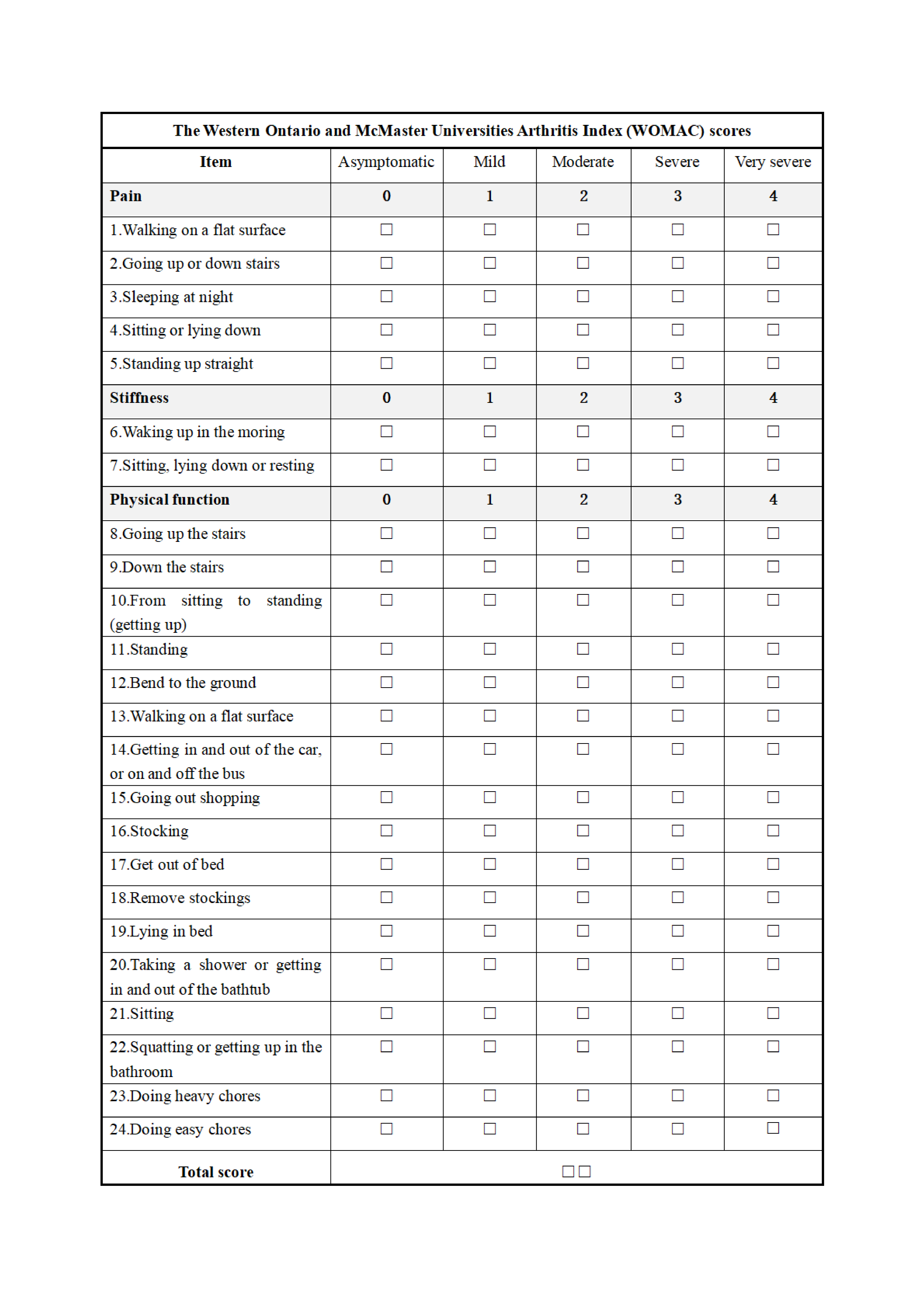

Supplement: Supplementary file 5 — Supplementary material 5. [file 13018_2024_4878_MOESM5_ESM.tiff]
